# Identification of a DNA methylation signature to predict disease-free survival in locally advanced rectal cancer

**DOI:** 10.18632/oncotarget.2347

**Published:** 2014-10-27

**Authors:** Jochen Gaedcke, Andreas Leha, Rainer Claus, Dieter Weichenhan, Klaus Jung, Julia Kitz, Marian Grade, Hendrik A. Wolff, Peter Jo, Jérôme Doyen, Jean-Pierre Gérard, Steven A. Johnsen, Christoph Plass, Tim Beißbarth, Michael Ghadimi

**Affiliations:** ^1^ Department of General and Visceral Surgery, University Medical Center Goettingen, Goettingen, Germany; ^2^ Department of Medical Statistics, University Medical Center Goettingen, Goettingen, Germany; ^3^ Division of Epigenomics and Cancer Risk Factors, German Cancer Research Center (DKFZ), Heidelberg, Germany; ^4^ Dept. of Medicine, Div. Hematology/Oncology, University of Freiburg Medical Center, Freiburg, Germany (present address); ^5^ Department of Pathology, University Medical Center Goettingen, Goettingen, Germany; ^6^ Department of Radiation Oncology, University Medical Center Goettingen, Goettingen, Germany; ^7^ Radiotherapy Department, Cyclotron Biomédical, Centre Antoine-Lacassagne Nice, France

## Abstract

In locally advanced rectal cancer a preoperative predictive biomarker is necessary to adjust treatment specifically for those patients expected to suffer relapse. We applied whole genome methylation CpG island array analyses to an initial set of patients (n=11) to identify differentially methylated regions (DMRs) that separate a good from a bad prognosis group. Using a quantitative high-resolution approach, candidate DMRs were first validated in a set of 61 patients (test set) and then confirmed DMRs were further validated in additional independent patient cohorts (n=71, n=42). We identified twenty highly discriminative DMRs and validated them in the test set using the MassARRAY technique. Ten DMRs could be confirmed which allowed separation into prognosis groups (p=0.0207, HR=4.09). The classifier was validated in two additional cohorts (n=71, p=0.0345, HR=3.57 and n=42, p=0.0113, HR=3.78). Interestingly, six of the ten DMRs represented regions close to the transcriptional start sites of genes which are also marked by the Polycomb Repressor Complex component EZH2. In conclusion we present a classifier comprising 10 DMRs which predicts patient prognosis with a high degree of accuracy. These data may now help to discriminate between patients that may respond better to standard treatments from those that may require alternative modalities.

## INTRODUCTION

Preoperative radiotherapy (RT) is standard in the treatment of locally advanced rectal cancer [1, 2]. Combining preoperative RT with 5-Fluorouracil (5-FU) increased local control [3] and resulted in a significantly decreased local recurrence rate compared to postoperative radiochemotherapy (CRT) [4]. However, even after ten years, successful reduction of local relapse has had no impact on disease-free (DFS) or overall survival [5]. Accordingly, the occurrence of distant metastases is a limiting factor, demonstrating the need of alternative treatments.

A significant proportion of patients receiving standard preoperative CRT responds to the therapy and does not experience distant metastases. Therefore, a more aggressive standard therapy for all patients would result in overtreatment of patients with an otherwise good prognosis whereby these patients would endure unnecessary toxicity. An a priori assessment of patients prior to therapy would therefore enable a risk-adapted approach. A similar strategy based on radiological assessment of high-risk patient groups was recently followed in the EXPERT-C trial [6].

Multifold molecular analyses have been applied to take advantage of the tumor biology in risk stratification or response prediction. However, the vast majority of analyses focused on single markers and failed to be validated in larger patient groups [7]. Interestingly, analyses of DNA methylation are rare, even though the regulation of transcriptional activity by methylation of cytosine residues is well known and constitutes a rather stable DNA modification [8, 9]. Moreover, changes in DNA methylation are also frequently associated with or indicative of other epigenetic changes, such as changes in histone modifications [10]. Furthermore, hypermethylation of specific regions is a frequent alteration observed in many different cancer types [11, 12].

Considering the complexity of DNA methylation changes in cancer, and in order to avoid missing the most promising regions, we performed an unbiased methylome analysis using a CpG island microarray. Secondly, using a matrix-assisted laser desorption/ionization (MALDI) time-of-flight (TOF) mass spectrometry-based (MS) technique (MassARRAY®), a subset of candidate prognosis-associated differentially methylated regions (DMRs) was assessed in a test set of patients (n=61). Selected DMRs were subsequently validated in a set of patients treated with preoperative CRT (n=71) as well as in a more heterogeneous set of patients treated with RT and CRT (n=42). The increased prognostic power of the CpG methylation signature compared to the previously analyzed CIMP was revealed.

## RESULTS

### Identification of DMRs

Genome-wide DNA methylation analysis was performed on 11 corresponding tumor and mucosa samples (screening set) using CpG island arrays. Data were analyzed for differentially methylated probes comparing good versus bad prognosis. Applying the above-mentioned criteria to DMRs, we identified a subset of 20 DMRs located within or close to 17 different genes (Supplementary Table 2). The majority of DMRs were distributed over chromosomes 1 (n=4), 19 (n=3), and 20q13 (n=5). This distribution could not be attributed to the frequency of array probes on the diverse chromosomes (p=7.6*10^−11^, Supplementary Figure 1).

### Target validation

To test the validity of the 20 identified DMRs, a group of 61 patients (test-set) was analyzed using MassARRAY®. Ten DMRs correlated significantly with DFS (p < 0.05). These DMRs were associated with genes *ADAP1*, *BARHL2*, *CABLES2*, *DOT1L*, *ERAS*, *ESRRG*, *RNF220*, *ST6GALNAC5*, *TAF4*, and *SLC20A2*. Unsupervised hierarchical clustering displayed a clear separation of patients into a high (n=31) or low methylation group (n=29) (Figure 2A). Log rank testing revealed a significantly better prognosis (p=0.0207, HR=4.09, 95%-CI=[1.12,14.87]) for the high methylation group (Figure 2B). An analysis of deviance comparing the full Cox model to that without methylation demonstrates a significantly better fit if methylation is part of the model (p=0.04). Analyzing for cancer-specific survival (CSS), only n=27 events occurred. Eleven DMRs significantly correlated with CSS, resulting in two patient groups. However, log rank testing failed to display any significant difference

**Figure 1:**
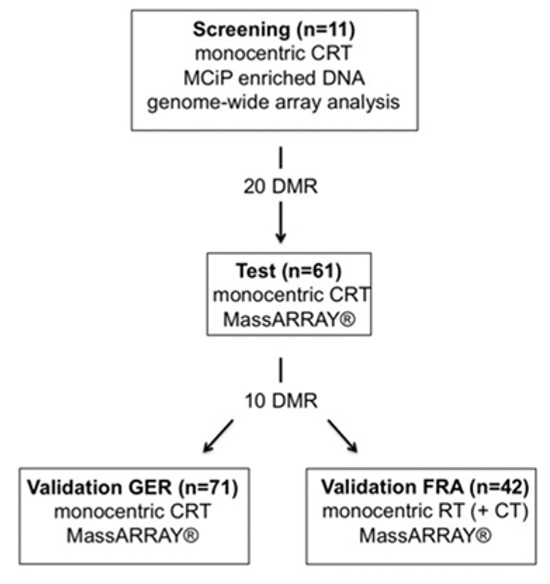
Study overview Study design comprising four different independent cohorts (MCIp - Methyl-CpG immunoprecipitation, DMR – differentially methylated region, MassARRAY® (technique by Sequenome) - matrix-assisted laser desorption/ionization (MALDI) time-of-flight (TOF) mass spectrometry (MS), CRT – chemoradiotherapy, RT – radiotherapy, GER – German cohort, FRA – French cohort)

**Figure 2:**
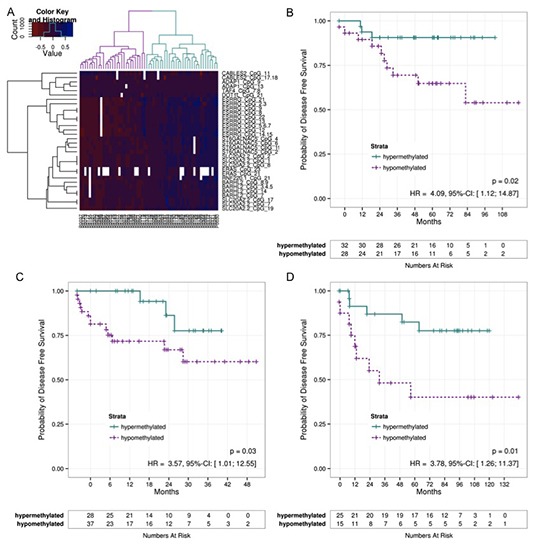
DFS of different patient cohorts **A)** Methylation sites measured in the test-Set were filtered for their correlation with DFS using a Cox regression. Based on the filtered CpGs a clustering was performed which is shown on the heatmap. The dendrogram on the columns illustrates a hyper- and a hyopmethylated group. **B)** The Kaplan-Meier estimates of these groups were compared using a log-rank test indicating a significant difference between hyper- and hypomethylated tumors indicating a good and a bad prognosis groups. The patients from the validation set GER **C)** and FRA **D)** were assigned to one of the clusters based on their methylation pattern by means of the nearest centroid distance. The Kaplan-Meier curves of the resulting patient groups were again compared using a log-rank test. Correlation to DFS again revealed significant differences. (DFS – Diseas Free Survival)

### Independent validation

Initial independent validation of the identified panel of 10 DMRs was performed on a set of 71 patients. Based on the methylation pattern of their tumor, patients were assigned to one of two clusters (good or bad prognosis). Comparing the survival curves of the resulting patient groups by means of a log-rank test, a significant difference (p=0.0345, HR=3.57, 95%-CI=[1.01, 12.55]) between both groups (Figure 2C) was again observed. Although comparison between the full model and that without methylation reveals a better fit for the full model, it was slightly over the 5% significance level (p=0.07).

A second validation of the 10 DMRs was applied to a more heterogeneous group of 42 patients [13]. After patients were assigned to a good or bad prognosis group a clear difference was observed (p=0.0113, HR=3.78, 95%-CI=[1.26,11.37]; Fig. 2D). Comparison between the full model and the model without methylation also results in a significantly better fit for the model with methylation (p=0.04).

### Comparison of CIMP and clinical data

To compare the newly identified gene set to our previous data on CIMP, we also applied the MassARRAY® technology to the specific CIMP panel but did not achieve statistical significance between the identified patient groups. To assess any further predictive value of these genes, both models were combined. However, the CIMP model did not add any additional predictive information compared to the whole genome model as illustrated in Supplementary Figure 2.

In addition to molecular analyses, preoperatively assessed clinical data are considered to have an influence on outcome. Accordingly, pretherapeutic tumor category or lymph node status were analyzed for any correlation to DFS using a univariate Cox regression model. However, both parameters failed to demonstrate any significant correlation (p=0.97 and p=0.08). Consequently, neither the CIMP genes nor the clinical data were used to complement the methylation profile identified from the CpG island methylome approach.

### Correlation of DMRs with Polycomb Repressor Complex occupancy

**Figure 3:**
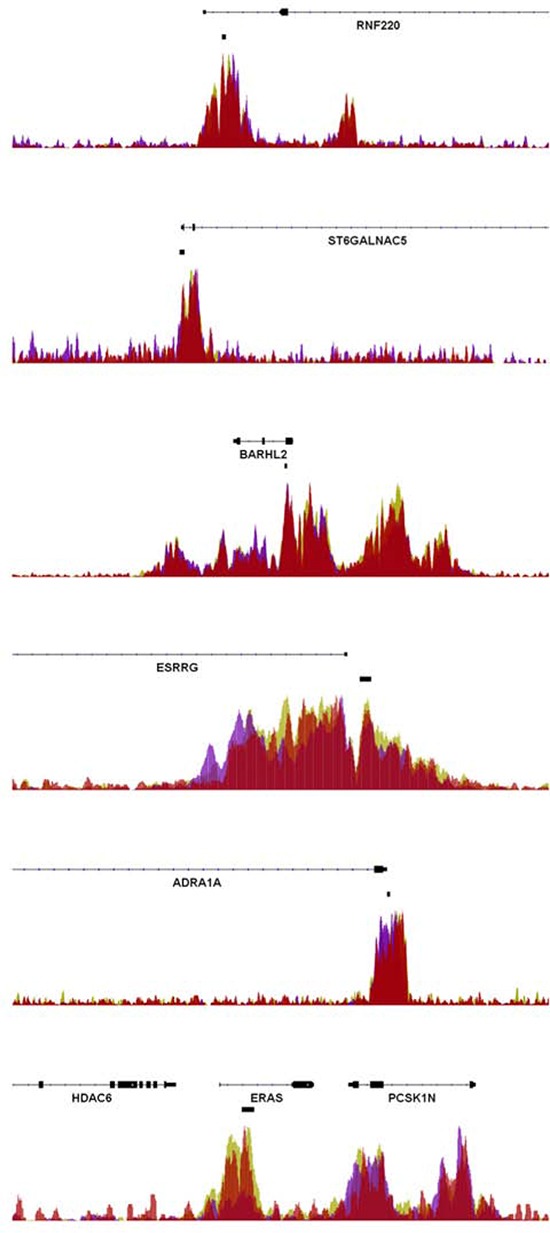
Enrichment of EZH2 at rectal cancer DMRs ChIP-seq data from H1 human embryonic stem cells, human umbilical vein endothelial cells, and normal human astrocytes obtained by the ENCODE consortium were analyzed for the occupancy of the Polycomb Repressor Complex-2 component EZH2 at the investigated rectal cancer-associated DMRs. As shown, significant signals of EZH2 occupancy were observed at the *RNF220*, *ST6GALNAC5*, *BARHL2*, *ESRRG*, *ADRA1A* and *ERAS* genes. The location of the DMRs is indicated by the black boxes located below the gene coordinates.

Like DNA methylation, a number of repressive post-translational histone modifications have been shown to be associated with tumorigenesis. Notably, a recent report suggested that a sequential epigenetic silencing process occurs during colorectal cancer tumor progression. In this model inactive genes are initially “poised” by being epigenetically marked by trimethylation of lysine 27 on histone H3 (H3K27me3) via the Polycomb Repressor Complex-2 component EZH2 [14]. The authors propose a model in which these genes become inactivated by subsequent DNA methylation during tumor progression. Therefore we utilized published chromatin immunoprecipitation sequencing (ChIP-seq) data in various cell types from the ENCODE consortium. Strikingly, six of the ten DMRs (near the *ADRA1A*, *BARHL2*, *ERAS*, *ESRRG*, *RNF220* and *ST6GALNAC5* genes) showed an enrichment of EZH2 (Figure 3).

## DISCUSSION

The differing response of patients with rectal cancer to preoperative radiochemotherapy as well as variable prognosis requires pre-therapeutic patient stratification. However, valid markers are still lacking. Consistent with our findings, analyzing pre-therapeutic clinical parameters such as localization or staging has failed to prove significant [15]. According to the EXPERT-C trial, the radiological assessment of the circumferential margin (CRM) has emerged as a possible parameter in the assessment of patients which has a high specificity [16]. However, radiological assessment of the CRM only identifies the localization of the tumor in relation to the mesorectal fascia and/or intersphincteric plane, but cannot assess the relation of the tumor to the final surgical boundary [17, 18]. Moreover, the definition disregards aggressive tumors that are small in size.

We therefore applied DNA methylation analyses to identify relevant methylation targets for the prediction of outcome in patients with advanced rectal cancer treated with CRT preoperatively. In an initial genome-wide screen on eleven patients, the 20 most representative DMRs were identified. These DMRs were preferentially and non-randomly distributed over chromosomes 1, 19, and 20q13.33, an area known for genetic variants associated with colorectal cancer [19, 20]. Linkage of DMRs suggests a common process responsible for aberrant methylation of the affected genomic regions which may involve the Polycomb Repressor Complex component EZH2. In a first set of patients, all 20 DMRs were tested for their ability to separate patients into a good or bad prognosis group. Within this test set, ten DMRs were significantly associated with DFS and clearly separated patients into a good or bad prognosis group. To further assess the predictive validity of these DMRs, they were validated in a set of 71 additional patients. The significant separation into a good or a bad prognosis group as identified by the Cox model underlined the predictive power of the retrieved methylation signature. In a second validation the signature was applied to a heterogeneous patient set that corresponds to a routine single-center trial. Patients were treated with radiotherapy only or in combination with 5-FU within previous clinical trials in France [3, 21].

It is important to acknowledge the treatment differences between the German and French cohorts with respect to preoperative therapy. The French cohort underwent primarily radiotherapy only with a small minority of patients receiving 5-FU in combination. Furthermore, the patients within the French cohort were not treated in an adjuvant setting. However, these patients were again separated into a good or bad prognosis group with respect to DFS. Accordingly, one may hypothesize that the signature is not restricted to a specific therapy, but rather allows a more generalized differentiation into more or less aggressive tumors. Therefore, it appears reasonable that each type of preoperative therapy may require a distinct methylation panel to predict the outcome precisely. Nevertheless, this needs to be analyzed on a larger patient cohort.

Interestingly, many of the identified DMRs within the signature are localized close to genes already associated with cancer, such as MEF2C and TAF4 [22]. *BARHL2*, a homebox gene, was found to be methylated in lung cancer [23] or astrocytomas [24]. *DOT1L* encodes a histone methyltransferase acting at lysine-79 of histone H3 [19] which was recently been shown to be an activator in colorectal cancer [25] and functions in DNA repair in yeast [26]. *ERAS* is epigenetically regulated and was shown to act as an oncogene by producing a constitutively active Ras protein [27]. Furthermore, its expression was revealed to mediate resistance to chemotherapy [28, 29]. *CABLES2* is involved in both p53-mediated and p53-independent apoptosis [30]. ST6GALNAC5, an enzyme which controls cell-cell or cell-extracellular matrix interactions [31] was also found in the signature. Other genes play a role in epigenetic regulation. ESRRG belongs to a receptor family functioning as a transcriptional activator of DNA cytosine-5-methyltransferases 1 (DNMT1) [20]. *Ring* finger protein 220 (RNF220) was shown to be a ubiquitin ligase targeting Sin3B thereby regulating the activity of the Sin3/HDAC corepressor complex [32]. Notably, many of these genes (*ADRA1A*, *BARHL2*, *ERAS*, *ESRRG*, *RNF220* and *ST6GALNAC5*) are also targets of the Polycomb Repressor Complex-2, further supporting an important role of epigenetic crosstalk in controlling rectal cancer therapeutic responsiveness.

While DNA methylation has already been analyzed in order to identify prognostic or predictive markers in rectal cancer in the past, these studies differed in their end-point and generally only analyzed smaller marker panels. Molinari et al. assessed the methylation status of 24 different tumor suppressor genes [33]. Except for the *TIMP3* gene no correlation to tumor regression grading and therefore the response to preoperative radiochemotherapy could be found. As a relevant endpoint for prognosis De Maat et al. focused on methylated- in-tumor (*MINT*) to assess the local recurrence rate [34]. Different methylation levels correlated to patient groups that were correlated to local recurrence. A large study by Kohonen-Corish et al. on 381 patients assessed *BRAF* and *KRAS* mutations, CIMP and microsatellite status as well as *CDKN2A* methylation [35]. *KRAS* mutation as well as *CDKN2A* methylation level was associated with prognosis and if found within the same tumor turned the prognosis was even worse. However, only 5% of the patients were treated preoperatively. Accordingly, these data cannot be compared. A recent study by Benard et al. analyzed 214 early rectal cancer patients that were also not treated by preoperative radiochemotherapy [36]. These showed a correlation between poor prognosis and hypomethylation of LINE-1 elements. Interestingly, Alu methylation status did not correlate with prognosis indicating a more specific role of LINE-1 with respect to prognosis compared with just the global methylation.

In colorectal cancer the CpG island methylator phenotype (CIMP) represents a distinct molecular subtype [37]. However, in the past different definitions of CIMP as well as technical variations have led to conflicting results as reviewed by Hughes et al. [38]. Moreover, the advantage of quantitative approaches over dichotomized values have been proposed by Claus et al. [39]. We therefore used the MassARRAY® technique to assess CIMP phenotype in rectal cancer patients. A small number of the analyzed CpGs from the *RUNX3*, *IGF2*, *SOCS1*, and *MINT1* genes showed a significant correlation between prognosis and methylation status, however, the number of patients considered as hypermethylated was small and the distinction between the two groups failed to show a clear correlation to disease free survival (Supplementary Figure 2). The small number of CIMP positive patients in rectal cancer has recently been reevaluated by Bae et al. [40] and a correlation between prognosis and CIMP status in the rectum was confirmed. Accordingly, CIMP should be considered as a small but relevant molecular marker in rectal cancer. With respect to previous data and the lack of concordance it should be acknowledged that the use of quantitative analyses led to a decreased number of patients in the CIMP positive group [39], resulting in an unbalanced analysis. Furthermore, the incomplete overlap between the DMRs assessed by MSP and MassArray likely contribute to these findings.

Aiming for individualized rectal cancer treatment, new trial designs based on the prediction of good or bad prognosis are needed. Accordingly, patients in the good prognosis group would receive standard, low-toxicity treatment. Patients assigned to a poor prognosis group should be subjected to intensified protocols at the cost of increased therapy-related toxicity. These regimens based on induction chemotherapy including antibody therapy before CRT [41, 42] have already been evaluated in the image-guided EXPERT-C trial [6] and displayed promising results with respect to prognosis. Certainly, the robustness of these data must be further analyzed. However, the methylation signature was reproducible despite the presence of a certain degree of patient heterogeneity. Patient cohorts differed by having a multicenter accrual and two validation sets, one from Germany and one from France. Nevertheless, prior to using these data for therapeutic decision making, they should be independently validated. As the different data sets have been retrieved by “all-in-one-batch-analyses” the assessment of methylation status “biopsy by biopsy” is another prerequisite. Furthermore, application of a more accessible technique and the reproducibility in FFPE samples are also relevant steps to assure the robustness.

In summary, we adopted a global methylome approach to identify potential targets that can predict outcome in advanced rectal cancer. Ten DMRs were identified and validated in two independent patient sets. This methylation panel represents the first validated global approach to pretherapeutic prediction in rectal cancer. If validated in a prospective trial, this signature could be applied to identify patients at high risk of developing distant metastases. Thus, stratification of preoperatively-treated rectal cancer patients using an individualized therapy approach based on molecular markers such as DMRs may indeed be achievable.

## MATERIAL AND METHODS

### Patients and treatment

Overall, 185 patients with preoperatively treated rectal cancer (0–12 cm from the anocutaneous verge) were included in the study (Table 1). One hundred and fifty-two patients were treated within or according to the CAO/ARO/AIO-94 or CAO/ARO/AIO-04 trial [4, 43]. Treatment was based on preoperative CRT comprising a total radiation dose of 50.4 Gy (single dose of 1.8 Gy) accompanied by either 5-FU or a combination of an intravenous infusion of oxaliplatin and 5-FU. Total mesorectal excision was performed 4 to 6 weeks after preoperative CRT. Patients were followed up according to the trial standards and provided written informed consent according to the guidelines set by the ethics committee of the University Medical Center Goettingen. Tumor and corresponding mucosa biopsies were taken during staging procedures prior to any therapy. A small set (n=11) was stored in liquid nitrogen while the vast majority was transferred to RNAlater (Ambion, Austin, TX) immediately after removal. Histopathologic work-up confirmed tumor tissue; a tumor-cell content of at least 50% was required for inclusion into the study. Forty-two samples from the “Lyon molecular signature” panel [13] were retrieved from patients who were treated in two randomized trials [3, 21]. These patients were treated differently and served as a validation panel. The dose of external beam radiation was 39 Gy (13×3 Gy) in the Lyon R 96 02 trial and 45 Gy in 25 fractions (1.8 Gy) over five weeks when included in FFCD 9203 trials. In the FFCD 9203 trial, patients were treated with concurrent 5-FU during the first and fifth week of external radiation. Biopsies taken prior to therapy were stored in liquid nitrogen without pathologic control [13].

**Table 1:** Clinical data of enrolled patients: Comparison of basic study parameters between test and validation sets Significant differences in the pre- and posttherapeutic therapy was due to the French cohort (GER – German cohort, FRA –French cohort, uT and uN – T-stage and lymph node status assessed by ultrasound, cM – clinically assessed distant metastases, 5-FU – 5-Fluorouracil, Ox – Oxaliplatin, RT – Radiotherapy, ypT and ypN – histopathologically assessed T-stage and lymph node status after preoperative therapy, yM – status of distant metastases after preoperative therapy, DFS – disease-free survival, CSS- cancer-specific survival)

Parameter	Pilot Set	Test Set	Validation Set (GER)	Validation Set (FRA)	adj. p value
**n**	11	61	71	42	
**Age [years]**					1.0
*mean ± sd*	66 ± 7.7	64 ± 11	63 ± 10	67 ± 10	
*median (min; max)*	65 (50; 77)	64 (36; 81)	63 (38; 80)	69 (42; 80)	
**Gender**					1.0
*female*	5 (45.5%)	18 (29.5%)	19 (26.8%)	18 (42.9%)	
*male*	6 (54.5%)	43 (70.5%)	52 (73.2%)	24 (57.1%)	
**uT**					1.0
*2*	0 (0.0%)	1 (1.6%)	3 (4.3%)	5 (11.9%)	
*3*	10 (90.9%)	57 (93.4%)	63 (90.0%)	36 (85.7%)	
*4*	1 (9.1%)	3 (4.9%)	4 (5.7%)	1 (2.4%)	
*NA*	0	0	1	0	
**uN**					1.0
*0*	1 (9.1%)	17 (27.9%)	24 (35.8%)	13 (31.0%)	
*1*	10 (90.9%)	44 (72.1%)	43 (64.2%)	29 (69.0%)	
*NA*	0	0	4	0	
**cM**					0.3
*0*	11 (100.0%)	60 (98.4%)	63 (88.7%)	42 (100.0%)	
*1*	0 (0.0%)	1 (1.6%)	8 (11.3%)	0 (0.0%)	
**Preoperative Therapy**					**0.01**
*5-FU + RT*	4 (36.4%)	31 (50.8%)	46 (64.8%)	6 (14.3%)	
*5-FU + Oxaliplatin + RT*	7 (63.6%)	30 (49.2%)	25 (35.2%)	0 (0.0%)	
*RT mono*	0 (0.0%)	0 (0.0%)	0 (0.0%)	36 (85.7%)	
**Surgery**					0.26
*APE*	1 (9.1%)	21 (34.4%)	13 (18.3%)	17 (40.5%)	
*LAR*	10 (90.9%)	40 (65.6%)	58 (81.7%)	25 (59.5%)	
**Postoperative Therapy**					**0.01**
*5-FU mono*	3 (27.3%)	28 (45.9%)	34 (50.7%)	6 (14.3%)	
*5-FU + Oxaliplatin*	7 (63.6%)	30 (49.2%)	23 (34.3%)	0 (0.0%)	
*none*	1 (9.1%)	3 (4.9%)	10 (14.9%)	36 (85.7%)	
*NA*	0	0	4	0	
**Cause of non- cancer-specific death**					1.0
*2nd malignancy*	1 (100.0%)	1 (14.3%)	0 (0.0%)	0 (0.0%)	
*heart failure*	0 (0.0%)	4 (57.1%)	1 (50.0%)	5 (100.0%)	
*Pneumonia*	0 (0.0%)	1 (14.3%)	0 (0.0%)	0 (0.0%)	
*pulmonary embolism*	0 (0.0%)	1 (14.3%)	0 (0.0%)	0 (0.0%)	
*surgical complication*	0 (0.0%)	0 (0.0%)	1 (50.0%)	0 (0.0%)	
**ypT**					1.0
*0*	4 (36.4%)	7 (11.5%)	15 (21.1%)	5 (12.5%)	
*1*	0 (0.0%)	8 (13.1%)	8 (11.3%)	2 (5.0%)	
*2*	2 (18.2%)	13 (21.3%)	15 (21.1%)	19 (47.5%)	
*3*	3 (27.3%)	31 (50.8%)	29 (40.8%)	14 (35.0%)	
*4*	2 (18.2%)	2 (3.3%)	4 (5.6%)	0 (0.0%)	
*NA*	0	0	0	2	
**ypN**					1.0
*negative*	9 (81.8%)	35 (57.4%)	49 (69.0%)	28 (70.0%)	
*positive*	2 (18.2%)	26 (42.6%)	22 (31.0%)	12 (30.0%)	
*NA*	0	0	0	2	
**yM**					1.0
*0*	11 (100.0%)	48 (78.7%)	55 (77.5%)	28 (66.7%)	
*1*	0 (0.0%)	13 (21.3%)	16 (22.5%)	14 (33.3%)	
**DFS probability**					0.29
*36-month estimate*	1	0.81	0.68	0.72	
*60-month estimate*		0.78		0.64	
**CSS probability**					1.0
*36-month estimate*		0.91	0.92	0.86	
*60-month estimate*		0.87		0.74	

The study design comprised a three-step procedure: first, a genome-wide CpG island screen for targets; second, testing of the most favorable targets; and third, a final validation (Figure 1). The screening and subsequent testing of identified regions was only performed on pretherapeutic biopsies of n=11 and n=61 patients treated in Goettingen. To validate these findings we utilized a German multicenter patient set (n=71) that was treated in an equivalent manner to the patients from the screening and test sets. Transferability of the identified signature was further validated with the n=42 patients from the “Lyon molecular signature” panel [13]. Due to the absence of an adequate follow-up but the need for fresh frozen biopsies to perform the genome-wide CpG scan, targets were selected based on tumor regression grading (TRG) and post-therapeutic lymph node status (ypN) after CRT. Both parameters are known to be well-established surrogate parameters for outcome in rectal cancer [15, 44–48]. The study was conducted in accordance with the Helsinki Declaration and was approved by the ethics committee of the University Medical Center Goettingen.

### DNA Isolation

For samples stored in liquid nitrogen, DNA isolation was performed using QIAamp DNA Mini Kit (Qiagen) according to the manufacturer's recommendations. In brief, frozen biopsies were disrupted using the TissueRuptor (Qiagen). Lysis was complemented with Proteinase K digest and DNA integrity was controlled by gel electrophoresis.

DNA from RNAlater samples was isolated using TRIZOL (Invitrogen, Carlsbad, CA) following standard procedures as previously described [49].

### MCIp enrichment and CpG island microarray analysis

MCIp enrichment of highly methylated DNA and CpG island microarray analysis was performed as described previously with minor modifications [50, 51]. Briefly, 2.5 µg DNA was sonicated with the Bioruptor NGS (Diagenode, Liege, Belgium) to fragments of 100 to 600 basepairs (bp). Fragmented DNA was MCIp-enriched using an SX-8G IP-Star robot (Diagenode) and 60 µg MBD2-Fc protein coupled to protein A magnetic beads (Diagenode). Proper DNA enrichment was monitored by quantitative real-time PCR targeting the imprinted gene *SNRPN*. The non-methylated allele elutes at low-salt, while the methylated allele elutes at high-salt concentration. Highly methylated tumor and healthy control DNA (matched normal mucosa from nearby the tumor) were labeled with Alexa Fluor 5 and 3, respectively, and cohybridized to a 244 K human CpG island microarray (Agilent, Germany, Böblingen) covering 27800 CpG islands represented by 199,399 probe sequences with a length of 45–60 bp per probe sequence.

### Quantitative DNA methylation (MassARRAY®)

Quantitative DNA methylation was assessed based on matrix-assisted laser desorption/ionization (MALDI) time-of-flight (TOF) mass spectrometry (MS) technique by Sequenom (referred to as MassARRAY® in the manuscript) as described previously [52]. Regions of interest as identified by MCIp analyses were targeted with PCR. Primers were derived from Sequenom's (Hamburg, Germany) EpiDesigner platform or the EpiPanel (Supplementary Table 1). Relation of methylation specific PCR (MSP) CIMP primers to MassARRAY® primers is shown in Supplementary Figure 2. After sodium bisulfite modification of genomic DNA (Zymoresearch, Freiburg, Germany), the target gene regions were amplified by PCR. Subsequently deoxynucleotides in the PCR reaction were inactivated by dephosphorylation using shrimp alkaline phosphatase. By tagging the reverse PCR primer with the T7 recognition sequence, a single-stranded RNA copy of the template was generated by *in vitro* transcription. After base specific (U-specific) cleavage by RNase A, the cleavage products were analyzed using MassARRAY®. Signals with a 16 Da shift are representative for methylation events, and signal intensity is correlated with the degree of DNA methylation.

### Statistics

CpG island array data were processed and normalized according to previous analyzes [53]. In short background was corrected and log2-ratio transformation was performed using the NormExp method with offset = 50 [54]. Intensity-based LOESS normalization on rank-invariant probes and negative controls was used to reduce variation between co-hybridized samples [55]. For normalization between arrays, log-intensity averages (A-values) and log-intensity ratios (M-values) were scaled to have the same median-absolute-value across the arrays. DMRs were defined using the following stepwise criteria: (i) a “region” in the genome which may become a DMR is a coherent stretch with more than one array probe and in which two vicinal probes are separated by ≤500 bp; (ii) per tumor sample (array), only the top 5% (i.e. 10,000) hypermethylated probes are considered; (iii) a DMR is represented by at least two vicinal top 5%-probes allowing a gap of a single non-top-5% probe; (iv) DMRs were chosen for further analysis if they were present in all, or at least in all except one representative arrays. Finally, the top five DMRs for either ypN positivity or negativity, and complete or non-responder were chosen, respectively. The methylation levels of CpGs in the test set were filtered according to their correlation with disease-free survival (DFS) by a Cox regression with the methylation level as explanatory variable. DFS was measured starting from surgery; local recurrence or metastases were counted as an event and non-cancer related death was censored. In this patient cohort, no isolated local recurrence (without the appearance of distant metastases) was diagnosed. The methylation sites with a resulting p-value of less then 5% were clustered. Samples with missing methylation information for more than 60% of the methylation were excluded from further analysis. The patients were then grouped according to the main branches resulting from this clustering and the Kaplan-Meier estimates of these groups were compared using a log-rank test. Two Cox models were fitted using age, gender, uN, and the clustering as explanatory variables: a full model and a model without the methylation clustering. The explanatory variables were chosen to include the (potentially) relevant parameters known at treatment onset and which demonstrate sufficient variation in the studied patient cohort. Both models were compared by means of an analysis of deviance, and a p value assessing the change in log-likelihood was calculated. The patients from the validation set were assigned to one of the clusters based on their methylation pattern by means of the nearest centroid distance, and the Kaplan-Meier curves of the resulting patient groups were again compared using a log-rank test. The measured methylation sites from the validation set were also correlated to DFS using a CpG-wise Cox regression and the overlap of the methylation sites with a resulting p-value of less than 5% to the identified DFS-related CpGs from the test set was determined. An analogous correlation analysis for DFS was also performed per DNA region with the averaged methylation level of the CpGs connected to each DNA region. All analyses were performed using R (version 2.15). To compare data sets from the CIMP panel and the whole genome approach, their ability (alone and combined) to classify into good or bad prognosis groups were compared. Subsequently, the classification results were implemented in a multivariate Cox model and tested in an analysis of variance.

### ChIP-seq data

Processed bigwig files for chromatin-immunopre cipitation sequencing (ChIP-seq) data for EZH2 from the ENCODE consortium [56] were downloaded from the UCSC Genome Browser website for H1 human embryonic stem cells (GSM1003524), human umbilical vein endothelial cells (HUVEC, GSM1003518) and normal human astrocytes (GSM1003532). Signal files were overlayed and viewed in the Integrated Genome Viewer (version 2.3).

## SUPPLEMENTARY FIGURES AND TABLES


