# Supplementary material for: Identification of a DNA methylation signature to predict disease-free survival in locally advanced rectal cancer
Source: Oncotarget. 2014 Oct 27;5(18):8123–35. doi: 10.18632/oncotarget.2347 (PMC4226671; doi:10.18632/oncotarget.2347)
Supplement: Supplementary file 1 [file oncotarget-05-8123-s001.pdf]

SUPPLEMENTARY FIGURES AND TABLES

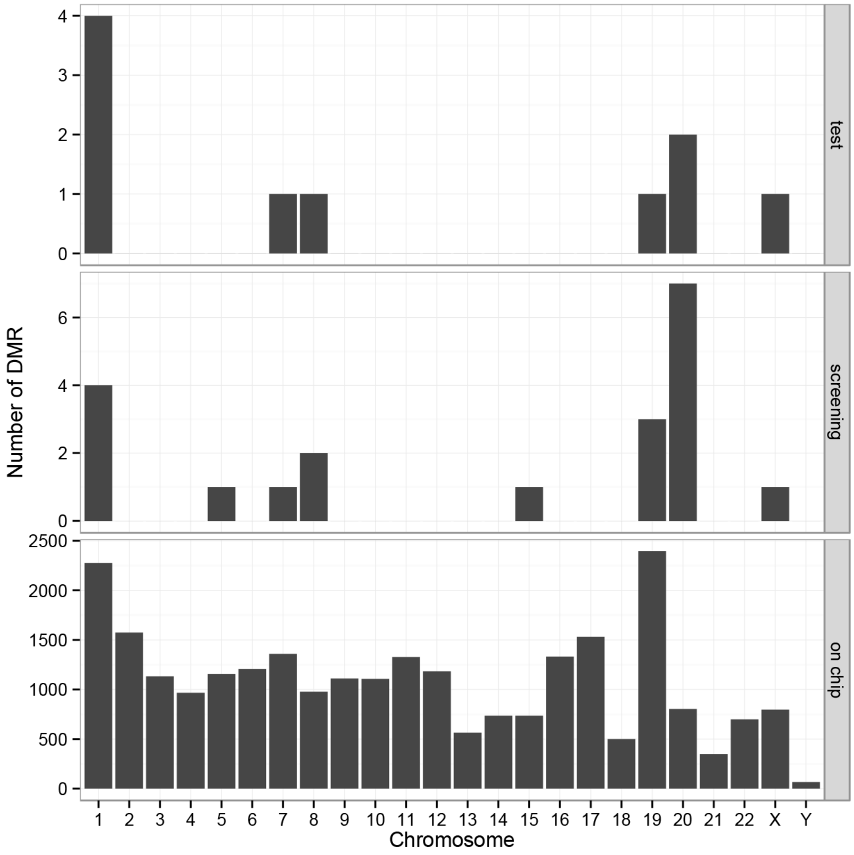

Supplementary Figure 1: DNA regions correlated to DFS in comparison with regions distributed on the methylation array.

A

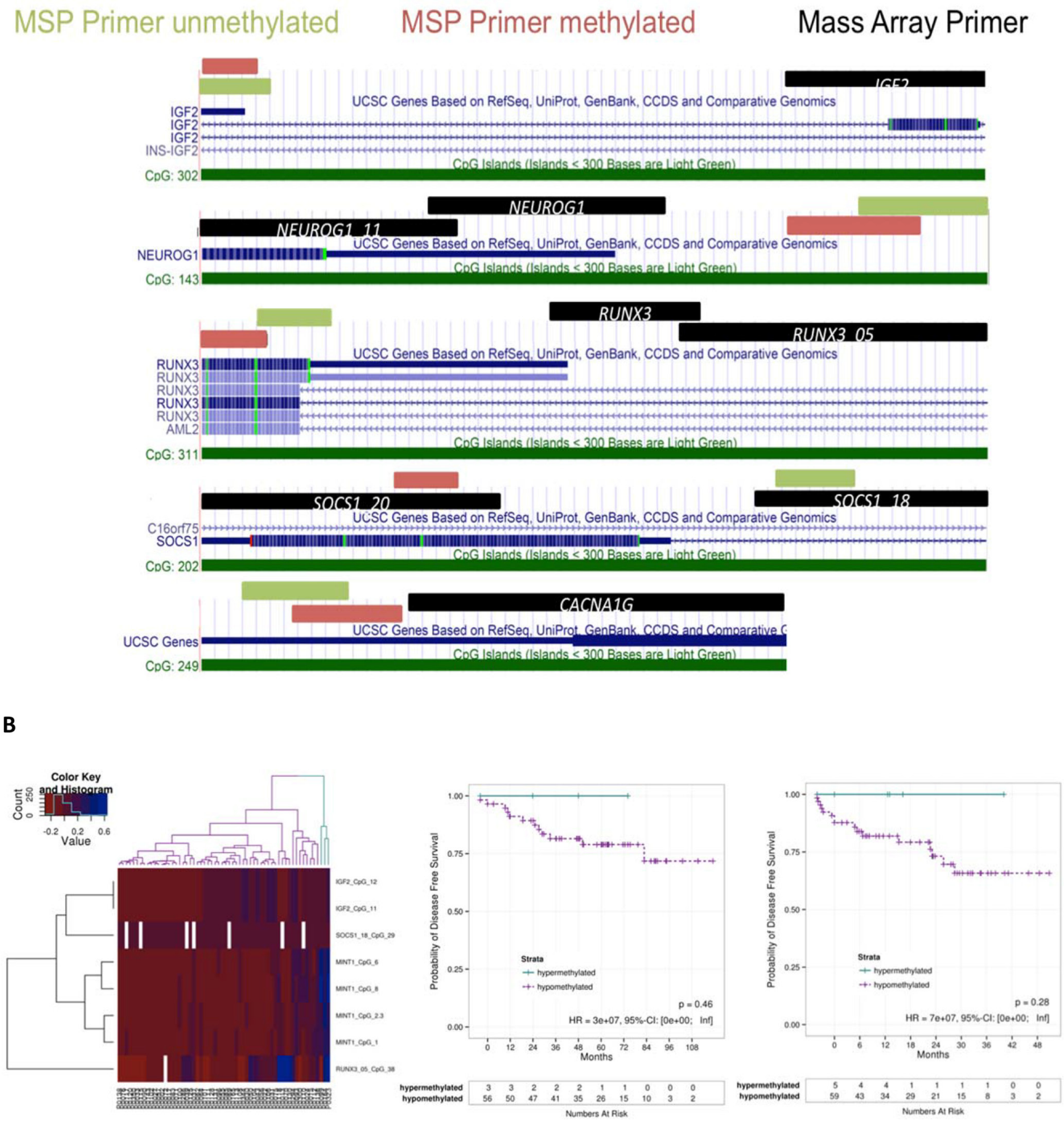

Supplementary Figure 2: Reevaluation of the CIMP using MassARRAY®

Supplementary Table 1. Primer sequences

Primer Name	Product Size (bp)	Sequence 5´-3´
CACNA1G00110F	410	aggaagagagTGTTTTGGTTTAAGTAGAAGAAAATTA
CACNA1G001T7R	410	cagtaatacgactcactatagggagaaggctAAAAAAACTAAACTAAACTAACCCTCCC
NEUROG110F	250	aggaagagagGGTATTTAAGGGGTTTTGAGGTTAG
NEUROG1T7R	250	cagtaatacgactcactatagggagaaggctTTCCCAACAACCTAAAATTATTACTC
RUNX310F	280	aggaagagagGGGTTTTGGGTTGTGGTATTG
RUNX3T7R	280	cagtaatacgactcactatagggagaaggctAAACAAATCCTCCAAAATCAAATAA
NEUROG11110F	300	aggaagagagGAGTAATAATTTTAGGTTGTTGGGAA
NEUROG111T7R	300	cagtaatacgactcactatagggagaaggctCCTATTAAAATCTAACACAATCTTCCTC
RUNX30510F	550	aggaagagagGGAGGAGGTTTTAGTGTTATAGTTTAG
RUNX305T7R	550	cagtaatacgactcactatagggagaaggctCCCCAAACTACTTAAAATCCC
SOCS11810F	400	aggaagagagGAGGGAGGGGAGTTTAGGGTAGTTAG
SOCS118T7R	400	cagtaatacgactcactatagggagaaggctTTCAACCTCAATAAACACAACTAAAAAAT
SOCS12010F	500	aggaagagagGGATTTTATTGGGGGTTTTTGAG
SOCS120T7R	500	cagtaatacgactcactatagggagaaggctACCCACATAATTCCAAACAAATAATA
MINT110F	300	aggaagagagGTAAGTGTTGAGAGAGTTTGAAAG
MINT1T7R	300	cagtaatacgactcactatagggagaaggctCCCCTCTAAACTTCACAACC
MINT210F	280	aggaagagagTTGATGTTTAATTGTAGGAAGG
MINT2T7R	280	cagtaatacgactcactatagggagaaggctAACTCCTCTAAAAACTTTCCAC
IGF210F	330	aggaagagagAATAGTATGGAGAGAAATAGAAAG
IGF2T7R	330	cagtaatacgactcactatagggagaaggctATCCAAATACAACCTCCACTC
ADAP110F	400	aggaagagagtatattatttttattaagaggaataag
ADAP1T7R	400	cagtaatacgactcactatagggagaaggctccaactctttccatcccc
ARFGAP1.110F	430	aggaagagagatagtaatatagattagagtttttgg
ARFGAP1.1T7R	430	cagtaatacgactcactatagggagaaggctatctccctaaaatcctacctcc
ARFGAP1.210F	350	aggaagagaggtatttttggggtttgggattag
ARFGAP1.2T7R	350	cagtaatacgactcactatagggagaaggctataaacctaataaacttaataataaaac
BARHL210F	260	aggaagagaggagattgggaattaggaggataag
BARHL2T7R	260	cagtaatacgactcactatagggagaaggctcatatacaccctacaaca
CABLES210F	320	aggaagagaggtttagatgtgtgtttggttttg
CABLES2T7R	320	cagtaatacgactcactatagggagaaggctaaaataacccttccctacacaac
CLEC4G10F	410	aggaagagagggtatagagaaaaagtagtaggag
CLEC4GT7R	410	cagtaatacgactcactatagggagaaggcttaaccctaaccccaacca
DOT1L10F	500	aggaagagagtgtgttttggaggggtttgttg
DOT1LT7R	500	cagtaatacgactcactatagggagaaggctaaatataaaaccctctccacttac
ERAS10F	480	aggaagagaggggtatttttataggatttttagaag
ERAST7R	480	cagtaatacgactcactatagggagaaggcttaacaacacatccccattcactc

(*Continued*)

Primer Name	Product Size (bp)	Sequence 5´-3´
ESRRG10F	480	aggaagagagttattgttttatgttttygggagag
ESRRGT7R	480	cagtaatacgactcactatagggagaaggctaaccattttactaataatttataaacc
GLTSCR110F	590	aggaagagagggagggtttagggtgtagtttg
GLTSCR1T7R	590	cagtaatacgactcactatagggagaaggctcccracttcctacttaatcttaaac
GMEB2.110F	500	aggaagagagggaggaaaygtgggatttgaag
GMEB2.1T7R	500	cagtaatacgactcactatagggagaaggcttaccccaacacaataaaaatccac
GMEB2.210F	450	aggaagagaggggtagattgggtgagtatttg
GMEB2.2T7R	450	cagtaatacgactcactatagggagaaggcttacaaaccctaaatcaacacaaa
MEF2C10F	480	aggaagagagaggttgttggtagggggtgag
MEF2CT7R	480	cagtaatacgactcactatagggagaaggctcattttacttaacccctaatattttc
NTSR110F	420	aggaagagagatttttagtaaggagtatataggtg
NTSR1T7R	420	cagtaatacgactcactatagggagaaggctccactaaaaactaaaacaataaacc
RNF22010F	370	aggaagagaggattagggtagttaggaggtag
RNF220T7R	370	cagtaatacgactcactatagggagaaggcttctcccctttacccctca
SLC20A2.110F	300	aggaagagaggaggtaggtagaaggagtttag
SLC20A2.1T7R	300	cagtaatacgactcactataggagaaggctccatcataaacctctacatcatc
SLC20A2.210F	400	aggaagagaggggaagatttagtattttgtatatg
SLC20A2.2T7R	400	cagtaatacgactcactatagggagaaggctaacacaacctctttccctaaaac
SNRPN10F	270	aggaagagagggattggaggatttggtgttg
SNRPNT7R	270	cagtaatacgactcactatagggagaaggctccttaaattaaaacaaaa
ST6GALNAC510F	500	aggaagagagtagaggtttttaggatttagtag
ST6GALNAC5T7R	500	cagtaatacgactcactatagggagaaggctaaaaattatccctttataacaaac
TAFA410F	300	aggaagagagggagttttagataaaagtagtaatg
TAFA4T7R	300	cagtaatacgactcactatagggagaaggctcctaactactctaattac

Supplementary Table 2. Analyzed DNA regions (category of prognosis refers to the genome-wide scan – DMR were identified based on prognosis groups from the surrogate parameters: postoperative nodal status and tumor regression grade; ypN pos – positive lymph node status after preoperative radiochemotherapy; ypn neg – negative lymph node status after preoperative radiochemotherapy; bad resp – bad response based on tumor regression grade, good resp – good response based on tumor regression grade; n.s. – not significant in the test-set)

Genes retrieved from genome-wide scan	Genes used for validation	Chrom.-location	Number of Studied CpGs	Category of prognosis
BARHL2	BARHL2	1p22.2	7	bad resp
ST6GALNAC5	ST6GALNAC5	1p31.1	15	ypN neg
RNF220	RNF220.1	1p34.1	12	bad resp
ESRRG	ESRRG	1q41	11	good resp
MEF2C	n.s.	5q14	15	ypN neg
ADAP1	ADAP1	7p22.3	13	good resp
SLC20A2.1	n.s.	8p11.21	7	good resp
SLC20A2.2	SLC20A2.2	8p11.21	12	good resp
SNRPN	n.s	15q11.2	7	bad resp
CLEC4G	n.s.	19p13.2	17	bad resp
DOT1L	DOT1L	19p13.3	18	ypN pos
GLTSCR1	n.s.	19q13.3	17	ypN pos
NTSR1	n.s.	20q13	10	good resp
ARFGAP1.1	n.s.	20q13.33	12	good resp
ARFGAP1.2	n.s.	20q13.33	8	good resp
CABLES2	CABLES2	20q13.33	12	good resp
GMEB2.1	n.s.	20q13.33	17	good resp
GMEB2.1	n.s.	20q13.33	14	good resp
TAF4	TAF4	20q13.33	8	good resp
ERAS	ERAS	Xp11.23	12	ypN pos
